# Secure two-party computation of solid triangle area and tetrahedral volume based on cloud platform

**DOI:** 10.1371/journal.pone.0217067

**Published:** 2019-06-13

**Authors:** Jing Zhang, Lixiang Li, Yongli Tang, Shoushan Luo, Yixian Yang, Yang Xin

**Affiliations:** 1 School of Computer and Information Technology, Beijing Jiaotong University, Beijing, China; 2 School of Computer Science and Technology, Henan Polytechnic University, Jiaozuo, Henan, China; 3 Information Security Center, National Engineering Laboratory for Disaster Backup and Recovery, Beijing University of Posts and Telecommunications, Beijing, China; Wuhan University, CHINA

## Abstract

With the emergence and widespread application of cloud computing, the use of cloud platforms to solve the problem of secure multi-party computation has emerged as a new research direction. The traditional computation of a solid geometry is performed through mutual interactions between two parties, which is not suitable in an untrusted cloud computing environment. In this paper, we first design a basic protocol for a secure Euclidean distance calculation that is suitable for cloud platforms and can serve as a building block for other protocols on cloud platforms. Using the solution of the Euclidean distance problem as such a building block, we provide a new method that converts the problems of calculating solid triangular areas and solid tetrahedral volumes into the calculation of distances and determinants in three-dimensional space. Then, we discuss solid point-line distance calculations, which extent the idea of the spatial geometry security problem. We present protocols for the above problems and prove that the proposed protocols can resist conspiracy among users and the untrusted cloud platform so that they can effectively ensure the privacy of the users. We also analyze the performances of these solutions. The analysis results show that our scheme is more versatile.

## Introduction

Secure multi-party computation is an important cryptographic primitive in the fields of modern cryptography and communication. In 1982, Yao et al. [1] raised the question of a millionaire. After in-depth research, Goldreich et al. theoretically proved that all SMC problems are solvable, and they proposed a universal solution [2] [3]. Subsequently, many researchers focused on the study of SMC and obtained a rich variety of research results [4] [5] [6] [7] [8] [9]. Some schemes have be used to solve problems such as electronic voting, organizing business flows, network data flows, position determination, and medical information [10] [11] [12] [13].

With the rise of cloud computing applications, increasingly more users want to entrust cloud platforms to perform intersection computing on private information. For example, Bob has two mixtures *ξ*_1_ and *ξ*_2_, which contain components *M*_1_, *M*_2_ and *M*_3_. Alice needs a new mixture, *ξ*_3_, that contains components *M*_1_, *M*_2_ and *M*_3_. With the cloud server, Alice wants to determine if she can produce this mixture from the two mixtures possessed by Bob. However, she does not want to disclose her needs. Similarly, Bob does not want to disclose the contents of his mixtures. This problem can be transformed into a secure two-party triangle area calculation problem based on cloud computing. Specifically, the participant uploads private data after processing to the cloud and then performs the computation required by the users with the cloud server. During the implementation, the cloud server does not obtain any private information of any participant. However, the cloud cannot be completely trusted because an adversary may perform improper actions through the cloud platform such as tampering with the sensitive data of the client or with the calculation results. How to perform computations concerning the allocation of mixtures with an untrusted cloud while protecting the user’s privacy is a challenging problem.

### Advantages of the scheme

In this paper, we move the traditional security computational geometry problem to the cloud platform, and we solve some solid geometry security problems. The main contributions of this study are as follows:

(1) Designing a solution for the secure multiparty computation of the square of the Euclidean distance (SMC-SED) with a cloud platform. In this scheme, Boneh encryption and blind factor are used to protect the user’s privacy. The benefit here is that the private user data participate in the subsequent calculation in the form of a ciphertext. Security and experimental analyses prove that our scheme can resist collusion between users and untrusted cloud platforms and is optimal. This solution is a building block and can be regarded as a new cloud computing technology.
(2) Focusing on the problem of triangular areas and that of tetrahedral volumes in three-dimensional space, we propose corresponding solutions, the Secure multiparty computation of triangle area calculation (SMC-TA) protocol and the Secure multiparty computation of tetrahedral volume calculation (SMC-TV) protocol. These new schemes convert the solid triangular area problem and the solid tetrahedral volume problem into the calculation of distances and determinants based on (1). Unlike traditional solutions, the cloud server is introduced as a third party to complete the core computing of the protocols. Even if an adversary were to break into the cloud server or compromise one of the users, he would not be able to obtain Bob’s private information. We prove the privacy-preserving property of these solutions using the security proof model, and we compare their performance with different schemes. The results show that our scheme is more versatile.
(3) Based on the secure multiparty computation solution for the triangle area computation, we solve the secure multiparty problem of the relationship between a point and a line and propose corresponding solutions. The security of this scheme is based on SMC-TA.

### Related work

#### The general SMC based on cloud services

Maheshwari et al. [14] proposed SMC solution techniques that could be embedded while designing a cloud computing architecture, especially when multiple cloud users jointly compute some function on their private data inputs. Kamara et al. [15] proposed the formalized definition of server-assisted secure multi-party computation, which required that there was no collusion between an ordinary participant and the server. On this basis, Kamara et al. [16] presented the concept of the secure function calculation of cloud auxiliary, and they constructed a protocol for the secure function calculation of a single server auxiliary. Carter et al. [17] proposed an implementation mechanism for private data protection in a cloud environment based on the outsourcing technology of Oblivious Transfer. This mechanism effectively mitigated dishonest behavior by cloud service providers. Compared to Yao’s chaotic circuit technology, the structure of SMC based on a cloud service is more secure given the use of homomorphic encryption techniques. Asharov et al. [18]constructed a protocol for SMC based on cloud services with a threshold homomorphic encryption scheme. In this protocol, participants are only required to execute calculations associated with the protocol, and the secure computation was performed by the cloud server. Lopez-alt et al. [19] proposed a complete multi-key homomorphic encryption scheme, and based on this scheme, they constructed the On-the-Fly Multi-party Computation (OFMC) protocol, which was secure under malicious adversaries.

#### The special SMC based on cloud services

Kerschbaum et al. [20] presented the non-interactive encrypted computation of the set intersection operation using an untrusted service provider. This service provider computed the intersection result after the users had submitted their encrypted sets to the service. The server could not obtain any information about the computation process. Kamara et al. [21] designed private set intersection (PSI) protocols in the server-aided setting, where the parties had access to a single untrusted server that made its computational resources available as a service. These protocols are secure in several adversarial models, and they address a range of security and privacy concerns such as fairness and leakage of the intersection size. Abadi et al. [22] designed a PSI on outsourced datasets based on a novel point-value polynomial representation, which ensured that intersections could only be calculated with the permission of all clients and that datasets and results remained completely confidential from the server. Veugen et al. [23] provided a generic framework that allowed an arbitrary number of users to securely outsource a computation to two non-colluding external servers with the help of a pre-processing phase that was independent of the inputs of the users. This approach was shown to be provably secure in an adversarial model. To address the inefficiency of previous schemes, Chen et al. [24] first transformed the original problem into a one-time evaluation problem for polynomials, and then, they designed four efficient and concise cloud computing environments to outsource the user set computing protocol. The analysis and comparison showed that these protocols were more efficient and concise than previously developed protocols. Chen et al. [25] transformed this traditional pattern into a cloud computation protocol that allowed an untrusted third party to be involved in the calculation process. They also designed a protocol for scalar product calculations applicable to cloud computing. On this basis, Chen the designed five solutions for spatial location relations. Although many researchers have begun to focus on SMC based on cloud services, fewer results on specific computing problems based on cloud services, especially for multi-party geometric computation, have been obtained. Research on such issues is attractive.

This paper focuses on some solid geometry problems based on cloud computing and their applications. We design the solution of secure multiparty computation of the square of the Euclidean distance using Boneh encryption. Using this solution as a building block, we solve the problem of triangular areas and that of tetrahedral volumes in three-dimensional space. On this basis, we give the protocol of the secure multiparty problem of the relationship between a point and a line.

## Preliminaries

We briefly review the groups underlying our scheme.

### Boneh encryption algorithm

Suppose that *E* is an encryption algorithm and that we are given the encryptions *C*_1_, *C*_2_ ∈ *G* of messages *m*_1_ and *m*_2_, respectively, where *C*_1_ = *E*(*m*_1_, *r*_1_) and *C*_2_ = *E*(*m*_2_, *r*_2_), in which *r*_1_, *r*_2_ are random numbers. We describe Boneh’s encryption algorithm and its homomorphic [26] as follows.

KeyGen (*τ*): Let *N* = *pq*, where *p*, *q* are two random primes. Generate a bilinear map *e*: (*G*_1_ × *G*_1_ → *G*), *G*_1_, *G* are groups with order *N* = *p* ⋅ *q*. Pick two random generators $g,u\overset{R}{\leftarrow }\;G$ and set *h* = *u*^*p*^. Then, *h* is a random generator of the subgroup of *G* with order *p*. The public key is *PK* = (*G*_1_, *G*, *e*, *g*, *h*, *N*), and the private key *SK* = *q*.

Encrypt (*PK*, *m*): Assume that the message space consists of integers in the set {0, 1, …, *n* − 1} with *T* < *q*_2_. To encrypt a message *m* (*m* < *p*) using the public key *PK*, pick a random number *r* ∈ *Z*_*N*_, compute *c* = *g*^*m*^ *h*^*r*^ ∈ *G* and output *c* as the ciphertext.

Decrypt(*SK*, *c*): To decrypt a ciphertext c using the private key *SK* = *q*_1_, observe that $c^{q_{1}}={\left(g^{m}\cdot h^{r}\right)}^{q_{1}}={{\left(g^{q_{1}}\right)}^{m}}^{}$. Let $g^{\prime }=g^{q_{1}}$. To recover *m*, it suffices to compute the discrete logarithm of $c^{q_{1}}$ base *g*′.

The Boneh encryption algorithm is clearly additively homomorphic. Suppose that *m*_1_ and *m*_2_ are messages. We have

$$\begin{array}{c} \begin{array}{cc} & E(m_{1})\cdot E(m_{\text{2}})=g^{m_{1}}h^{r_{1}}\cdot g^{m_{2}}\cdot h^{r_{2}}=g^{m_{1}+m_{2}}\cdot h^{r_{1}+r_{2}}\\ & =g^{m_{1}+m_{2}}\cdot h^{r}=E(m_{1}+m_{2},r). \end{array} \end{array}$$

Anyone can create a uniformly distributed encryption of *m*_1_ + *m*_2_ by the above formula for a random *r*.

More importantly, anyone can multiply two encrypted messages once using the bilinear map. Set *e*(*g*, *g*) = *g*, *e*(*g*, *h*) = *h*_1_, write *h* = *g*^*αp*^ for some (unknown) *α*, and pick a random *r*; then, we obtain

$$\begin{array}{c} \begin{array}{cc} & e(E(m_{1}),E(m_{2}))\\ = & e(g^{m_{1}}\cdot h^{r_{1}},g^{m_{2}}\cdot h^{r_{2}})\cdot {h_{1}}^{r}\\ = & e{\left(g,g\right)}^{m_{1}m_{2}}\cdot e{{\left(g,h\right)}^{m_{2}r_{1}+m_{1}r_{2}}}^{+\alpha pr_{1}r_{2}}\cdot {h_{1}}^{r}\\ = & g^{m_{1}m_{2}}\cdot {h_{1}}^{r^{\prime }} \end{array} \end{array}$$

where *r*′ = *m*_2_*r*_1_ + *m*_1_*r*_2_ + *αpr*_1_*r*_2_+r is distributed uniformly.

### Cayley-Menger determinant

The Cayley-Menger determinant is often used to address the Euclidean distance problem of an invariant space. This plays a fundamental role in the so-called “distance geometry” [27]. The Cayley-Menger bideterminant of two sequences with *n* vectors (*p*_1_, *p*_2_, …*p*_*n*_) and (*q*_1_, *q*_2_, …, *q*_*n*_) ∈ *R*^*m*^ is defined as

$$\begin{array}{c} \begin{array}{cc} & D(p_{1},p_{2},...p_{n};q_{1},q_{2},...,q_{n})\\ & =2{\left(\frac{-1}{2}\right)}^{n}|\begin{array}{ccccc} 0 & 1 & 1 & ... & 1\\ 1 & D(p_{1},q_{1}) & D(p_{1},q_{2}) & ... & D(p_{1},q_{n})\\ 1 & D(p_{2},q_{1}) & D(p_{2},q_{2}) & ... & D(p_{2},q_{n})\\ ... & ... & ... & ... & ...\\ 1 & D(p_{n},q_{1}) & D(p_{n},q_{2}) & ... & D(p_{n},q_{n}) \end{array}| \end{array} \end{array}$$

where *D*(*p*_*k*_, *q*_*l*_) denotes the squared distance between *p*_*k*_ and *q*_*l*_. In many cases, these two sequences are the same. Hence, it will be convenient to abbreviate *D*(*p*_1_, *p*_2_, …*p*_*n*_; *q*_1_, *q*_2_, …, *q*_*n*_) by *D*(*p*_1_, *p*_2_, …*p*_*n*_), which is simply called a Cayley-Menger determinant.

In *m*-dimensional Euclidean space, the rank of the matrix of Cayley-Menger determinants is no greater than *m* + 1. Specifically, *D*(*p*_1_, *p*_2_, …*p*_*n*_) = 0 when *n* >= *m* + 1. Let us show the geometric interpretation of Cayley-Menger determinants when *n* = 2, 3, 4 and *m* = 3.

For *n* = 2,

$$\begin{array}{c} D(p_{1},p_{2})=d{\left(p_{1},p_{2}\right)}^{2} \end{array}$$

where *D*(*p*_1_, *p*_2_) is the Euclidean distance between *p*_1_ and *p*_2_. Observe that the squared distance between *p*_1_ and *p*_2_ is consistent with the result of the Cayley-Menger determinant.

For *n* = 3,

$$\begin{array}{c} \begin{array}{cc} D(p_{1},p_{2},p_{3}) & =4A^{2}={∥\left(p_{2}-p_{1})\times (p_{3}-p_{1}\right)∥}^{2}\\ & =2\cdot {\left(\frac{-1}{2}\right)}^{3}|\begin{array}{cccc} 0 & 1 & 1 & 1\\ 1 & 0 & {L^{2}}_{12} & {L^{2}}_{13}\\ 1 & {L^{2}}_{12} & 0 & {L^{2}}_{23}\\ 1 & {L^{2}}_{12} & {L^{2}}_{23} & 0 \end{array}| \end{array} \end{array}$$

where *A* is the area of the triangle spanned by *p*_1_, *p*_2_, *p*_3_, and ‖‖ is the length of vector. *L*_*ij*_ is the distance between *p*_*i*_ and *p*_*j*_, *i*, *j* = 1, 2, 3.

For *n* = 4,

$$\begin{array}{c} \begin{array}{cc} & D(p_{1},p_{2},p_{3},p_{4})=36V^{2}\\ & =2\cdot {\left(\frac{-1}{2}\right)}^{4}\cdot |\begin{array}{ccccc} 0 & 1\;\;\;\;\;\;\; & 1\;\;\;\;\;\;\; & 1\;\;\;\;\;\;\;\;\; & 1\;\;\;\;\;\;\\ 1 & 0\;\;\;\;\;\;\; & {L^{2}}_{12} & {L^{2}}_{13} & {L^{2}}_{14}\\ 1 & {L^{2}}_{12} & 0\;\;\;\;\;\;\;\;\; & {L^{2}}_{23} & {L^{2}}_{24}\\ 1 & {L^{2}}_{13} & {L^{2}}_{23} & 0\;\;\;\;\;\;\;\;\;\; & {L^{2}}_{34}\\ 1 & {L^{2}}_{13} & {L^{2}}_{24} & {L^{2}}_{34} & 0\;\;\;\;\;\;\;\;\;\; \end{array}| \end{array} \end{array}$$

where *V* is the volume of the tetrahedron spanned by *p*_1_, *p*_2_, *p*_3_, *p*_4_, and *L*_*ij*_ is the distance between *p*_*i*_ and *p*_*j*_, *i*, *j* = 1, 2, 3, 4.

### Security proof in the semi-honest model

In the semi-honest model, the parties abide by the protocol. However, they keep a record of all the intermediate computations and expect to deduce the private inputs of other parties from the record. The security of secure two-party computations in the semi-honest model can be described as follows. There is a probability polynomial-time algorithm (referred to as a simulator). Using this simulator, any semi-honest participant can simulate the execution process of the protocol alone and obtain all the intermediate information using his own inputs and the final result from the protocol.

**Definition 1** (The security of secure two-party computations in the semi-honest model) Let *f*: {0, 1}* × {0, 1}* → {0, 1}* × {0, 1}* be a functionality, where *f*_*i*_(*x*, *y*), *i* = 1, 2 is the *i*th element of *f*(*x*, *y*) and Π is a two-party protocol for computing *f* (denoted as *f*^2^). The view of the *i*th party during an execution of Π on (*x*, *y*), denoted as $VIEW_{i}^{\Pi }(x,y)$, is (*x*, *r*, *m*_1_, …, *m*_*t*_), where *r* represents the outcome of the *i*th party’s internal coin tosses, and *m*_*j*_ represents the *j*th message that the *i*th party has received. Let $OUTPUT_{i}^{\Pi }(x,y)$ be the *i*th output result. If there exists a probabilistic polynomial-time algorithm, denoted as *S*_1_ and *S*_2_, making (1) and (2) workable, we say that Π privately computes *f*.

$$\begin{array}{c} \begin{array}{ccc} & {\left\{S_{1}(x,f_{1}(x,y))\right\}}_{x,y\in {\left\{0,1\right\}}^{*}s.t.|x|=|y|}\overset{C}{\equiv } & {\left\{VIEW_{1}^{\Pi }(x,y)\right\}}_{x,y\in {\left\{0,1\right\}}^{*}s.t.|x|=|y|} \end{array} \end{array}$$ (1)

$$\begin{array}{c} \begin{array}{ccc} & {\left\{S_{2}(y,f_{2}(x,y))\right\}}_{x,y\in {\left\{0,1\right\}}^{*}s.t.|x|=|y|}\overset{C}{\equiv } & {\left\{VIEW_{2}^{\Pi }(x,y)\right\}}_{x,y\in {\left\{0,1\right\}}^{*}s.t.|x|=|y|} \end{array} \end{array}$$ (2)

### Problems

This paper studies the following problems.

Problem 1: Secure multiparty computation of the square of the Euclidean distance(SMC-SED). Alice has a private point *P*_*A*_ = (*x*_1_, *x*_2_, …, *x*_*n*_), and Bob has a private point *P*_*B*_ = (*y*_1_, *y*_2_, …, *y*_*n*_), where (*n* ≥ 3). Alice and Bob want to know the Euclidean distance between the points *P*_*A*_ = (*x*_1_, *x*_2_, …, *x*_*n*_) and *P*_*B*_ = (*y*_1_, *y*_2_, …, *y*_*n*_), where (*n* ≥ 3), denoted by *D*^2^(*P*_*A*_, *P*_*B*_), without disclosing *P*_*A*_ or *P*_*B*_.
Problem 2: Secure multiparty computation of the area of a triangle(SMC-TA). Alice has a private point *P*_*A*_ = (*x*_*A*_, *y*_*A*_, *z*_*A*_), and Bob has private two points $P_{B_{1}}=(x_{B_{1}},y_{B_{1}},z_{B_{1}})$ and $P_{B_{2}}=(x_{B_{2}},y_{B_{2}},z_{B_{2}})$. Alice and Bob want to know the area of the triangle formed by the points *P*_*A*_ = (*x*_*A*_, *y*_*A*_, *z*_*A*_), $P_{B_{1}}=(x_{B_{1}},y_{B_{1}},z_{B_{1}})$, $P_{B_{2}}=(x_{B_{2}},y_{B_{2}},z_{B_{2}})$, denoted by $S_{\Delta AB_{1}B_{2}}$, without disclosing *P*_*A*_, $P_{B_{1}}$ or $P_{B_{2}}$.
Problem 3: Secure multiparty computation of the volume of a tetrahedron(SMC-TV). Alice has a private point *P*_*A*_ = (*x*_*A*_, *y*_*A*_, *z*_*A*_), and Bob has two private points $P_{B_{1}}=(x_{B_{1}},y_{B_{1}},z_{B_{1}})$, $P_{B_{2}}=(x_{B_{2}},y_{B_{2}},z_{B_{2}})$, and $P_{B_{3}}=(x_{B_{3}},y_{B_{3}},z_{B_{3}})$. Alice and Bob want to know the volume of the tetrahedron formed by the points *P*_*A*_ = (*x*_*A*_, *y*_*A*_, *z*_*A*_), $P_{B_{1}}=(x_{B_{1}},y_{B_{1}},z_{B_{1}})$, $P_{B_{2}}=(x_{B_{2}},y_{B_{2}},z_{B_{2}})$, and $P_{B_{3}}=(x_{B_{3}},y_{B_{3}},z_{B_{3}})$ denoted by $V_{AB_{1}B_{2}B_{3}}$, without disclosing *P*_*A*_, $P_{B_{1}}$,$P_{B_{2}}$, or $P_{B_{3}}$.
Problem 4: The distance between a point and a line(SMC-DPL). Alice has a private point *P*_*A*_ = (*x*_*A*_, *y*_*A*_, *z*_*A*_), and Bob has a private line
  $$\begin{array}{c} L_{}:\{\begin{array}{c} A_{1}x+B_{1}y+C_{1}z+D_{1}=0\\ A_{2}x+B_{2}y+C_{2}z+D_{2}=0 \end{array} \end{array}$$
  Alice and Bob want to know the distance between the point *P*_*A*_ and the line *L* without disclosing *P*_*A*_ and *L*.

In the next sections, we will give our solutions to the four problems in detail.

## Building block

Alice has an *n*-dimensional point *P*_*A*_ = (*x*_1_, *x*_2_, …, *x*_*n*_), and Bob has an *n*-dimensional point *P*_*B*_ = (*y*_1_, *y*_2_, …, *y*_*n*_), where *n* ≥ 3. Alice and Bob want to compute the Euclidean distance without disclosing the messages of their points. We call this problem Secure multiparty computation of the square of Euclidean distance (SMC-SED). This problem is the building block of the other three problems. To solve this problem, we propose a protocol for the secure multiparty computation of the square of the Euclidean distance with the Boneh encryption algorithm. The solution transfers the main calculation to the cloud, which makes it possible for users to only perform encryption and other simple operations.

### The solution of SMC-SED

**Fig 1:**
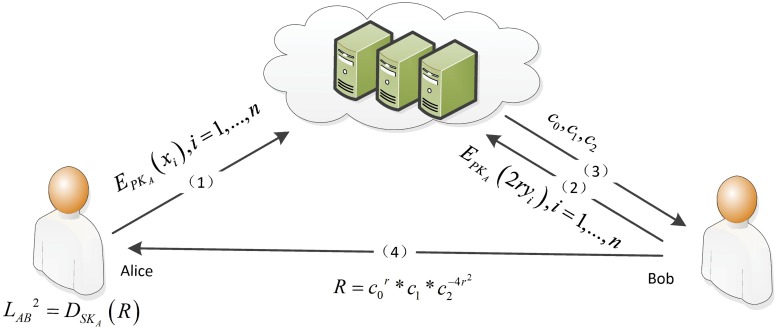
The solution of SMC-SED.

The protocol for computing the square of the Euclidean distance is depicted in Fig 1. First, Alice and Bob encrypt their private values and then send their values to the server. To do so, Bob’s values are blinded so that the server cannot determine them, even if it colludes with Alice. Then, the server uses the Boneh algorithm to calculate *c*_0_, *c*_1_, and *c*_2_ and sends them to Bob. In the third step, Bob eliminates the blinding factors (*r* in the protocol and computes the cipher values. Therefore, Bob cannot decrypt the obtained information in this step. At the end of the protocol, Alice decrypts the cipher values and receives the square of the Euclidean distance. The protocol is described as follows.

**Protocol 1**. SMC-SED

**Inputs**: Alice’s input is *P*_*A*_ = (*x*_1_, *x*_2_, …, *x*_*n*_). Bob’s input is *P*_*B*_ = (*y*_1_, *y*_2_, …, *y*_*n*_), where (*n* ≥ 3).

**Outputs**: The square of the Euclidean distance formed by *P*_*A*_, *P*_*B*_.

Step 1 Based on the Boneh encryption algorithm, Alice picks suitable parameters and generates the public-private key pair (*PK*_*A*_, *SK*_*A*_);
Step 2 Alice computes $E_{PK_{A}}(x_{i})$, *i* = 1…*n* and sends it to the cloud server;
Step 3 Bob picks a random number *r*(≠ 0) and computes $E_{PK_{A}}(2ry_{i})$, where *i* = 1…*n*. Then, Bob sends $E_{PK_{A}}(2ry_{i})$ to the cloud server;
Step 4 The cloud server obtains the messages that originated from Alice and Bob, and it performs the following calculations with the homomorphic property of the Boneh encryption algorithm:
  $$\begin{array}{c} \begin{array}{cc} c_{0} & =e(E_{PK_{A}}(x_{1}),E_{PK_{A}}(2ry_{1}))...e(E_{PK_{A}}(x_{n}),E_{PK_{A}}(2ry_{n}))\\ & =E(2rx_{1}y_{1}+2rx_{2}y_{2}+...+2rx_{n}y_{n})\\ & =E{\left(-2x_{1}y_{1}-2x_{2}y_{2}-...-2x_{n}y_{n}\right)}^{-r} \end{array} \end{array}$$
  $$\begin{array}{c} \begin{array}{cc} c_{1} & =e(E_{PK_{A}}(x_{1}),E_{PK_{A}}(x_{1}))...e(E_{PK_{A}}(x_{n}),E_{PK_{A}}(x_{n}))\\ & =E_{PK_{A}}(x_{1}^{2}+...+x_{n}^{2}) \end{array} \end{array}$$
  $$\begin{array}{c} \begin{array}{cc} c_{2} & =e(E_{PK_{A}}(2ry_{1}),E_{PK_{A}}(2ry_{1}))...e(E_{PK_{A}}(2ry_{n}),E_{PK_{A}}(2ry_{n}))\\ & =E_{PK_{A}}(4r^{2}(y_{1}^{2}+...+y_{n}^{2}))\\ & =E_{PK_{A}}{\left(y_{1}^{2}+...+y_{n}^{2}\right)}^{4r^{2}} \end{array} \end{array}$$
  Then, the cloud server sends *c*_0_, *c*_1_, *c*_2_ to Bob;
Step 5 Bob computes $R={c_{0}}^{r}*c_{1}*c_{2}^{-4r^{2}}$ and sends *R* to Alice;
Step 6 Alice computes ${L_{AB}}^{2}=D_{SK_{A}}(R)$ and tells the result to Bob.

### Security

As we know, the cloud sever is untrusted. Therefore, we have to consider the security of private information in the cloud and the complicity between the cloud and any participant. Therefore, the security model of the protocol is slightly different than the traditional model. This will be proved with five formulas in Table 1. [25]

**Table 1:** The security of the protocol based on the cloud platform.

Type	Privacy	Simulation
*No collusion*	Bob’s privacy	$\left\{view_{1}^{\Pi }(P_{A,}P_{B})\}\overset{C}{\equiv }\{S_{1}(P_{A},f_{1}(P_{A,}P_{B}))\right\}$
*No collusion*	Alice’s privacy	$\left\{view_{2}^{\Pi }(P_{A,}P_{B})\}\overset{C}{\equiv }\{S_{2}(P_{B},f_{2}(P_{A,}P_{B}))\right\}$
*No collusion*	Alice’s privacy and Bob’s privacy	$\;\{view_{3}^{\Pi }(P_{A},P_{B})\}\;\;\;\overset{C}{\equiv }\;\;\;\;\{S_{3}(E(P_{A}),E(P_{B}),E(P_{A},P_{B}))\}$
*Collusion*	Bob’s privacy (Server and Alice collusion)	$\left\{view_{4}^{\Pi }(P_{A},P_{B})\}\;\overset{C}{\equiv }\{S_{4}(P_{A},E(P_{A})E(P_{B}),f_{1}(P_{A,}P_{B}),E(P_{A},P_{B}))\right\}$
*Collusion*	Alice’s privacy (Server and Bob collusion)	$\left\{view_{5}^{\Pi }(P_{A},P_{B})\}\overset{C}{\equiv }\{S_{5}(P_{B},E(P_{A})E(P_{B}),f_{2}(P_{A,}P_{B}),E(P_{A},P_{B}))\right\}$

Theorem 1. The SMC-SED protocol, denoted by Π, is private, where *n* ≥ 3.

Proof. We will prove this theorem by showing five simulators *S*_1_, *S*_2_, *S*_3_, *S*_4_, *S*_5_.

(1) We first show the construction of *S*_1_. Based on the inputs *P*_*A*_ = (*x*_1_, *x*_2_, …, *x*_*n*_) and *L*_*AB*_^2^, *S*_1_ proceeds as follows.

1. *S*_1_ computes $E_{PK_{A}}(x_{i}),i=1,...,n$. Then, *S*_1_ chooses a point *P*_*B*_′ = (*y*_1_′, …, *y*_*n*_′) and a random number *r*′. It computes $E_{PK_{A}}(2r^{\prime }{y^{\prime }}_{i})$, where *i* = 1, …, *n*;
2. *S*_1_ obtains $E_{PK_{A}}(x_{i})$, *P*_*B*_′ = (*y*_1_′, *y*_2_′, …, *y*_*n*_′), where *i* = 1, 2, 3…*n*, and performs the following calculations with the homomorphic property of the Boneh encryption algorithm:
  $$\begin{array}{c} \begin{array}{cc} {c_{0}}^{\prime } & =e(E_{PK_{A}}(x_{1}),E_{PK_{A}}(2r^{\prime }y_{1}^{\prime }))...e(E_{PK_{A}}(x_{n}),E_{PK_{A}}(2r^{\prime }y_{n}^{\prime }))\\ & =E{\left(-2x_{1}y_{1}^{\prime }-2x_{2}y_{2}^{\prime }-...-2x_{n}y_{n}^{\prime }\right)}^{-r^{\prime }} \end{array} \end{array}$$
  $$\begin{array}{c} \begin{array}{cc} c_{1} & =e(E_{PK_{A}}(x_{1}),E_{PK_{A}}(x_{1}))...e(E_{PK_{A}}(x_{n}),E_{PK_{A}}(x_{n}))\\ & =E_{PK_{A}}(x_{1}^{2}+...+x_{n}^{2}) \end{array} \end{array}$$
  $$\begin{array}{c} \begin{array}{cc} {c_{2}}^{\prime } & =e(E_{PK_{A}}(2r^{\prime }y_{1}^{\prime }),E_{PK_{A}}(2r^{\prime }y_{1}^{\prime }))...e(E_{PK_{A}}(2r^{\prime }{y^{\prime }}_{n}),E_{PK_{A}}(2r^{\prime }{y^{\prime }}_{n}))\\ & =E_{PK_{A}}{\left({y_{1}^{\prime }}^{2}+...+{y_{n}^{\prime }}^{2}\right)}^{4r^{\prime 2}} \end{array} \end{array}$$
  Then, *S*_1_ computes $R^{\prime }=c_{0}^{\prime }{}^{-r^{\prime }}*c_{1}*{c_{2}^{\prime }}^{-4\cdot {r^{\prime }}^{2}};$
3. *S*_1_ decrypts the result $L_{AB}{{}^{\prime }}^{2}={\text{D}}_{SK_{A}}(R^{\prime })$;
4. *S*_1_ outputs the message list of Alice:
  $$\begin{array}{c} \begin{array}{c} \;\{S_{1}(P_{A},f_{A}(P_{A,}P_{B}))\}=\{(SK_{A},PK_{A}),P_{A},E_{PK_{A}}(P_{A}),R^{\prime },L_{AB}{{}^{\prime }}^{2}\} \end{array} \end{array}$$
  Note that in this protocol,
  $$\begin{array}{cc} \;view_{1}^{\Pi }(P_{A},P_{B})= & \left\{(SK_{A},PK_{A}),P_{A},E_{PK_{A}}(P_{A}),R,{L_{AB}}^{2}\right\}\\ & f_{1}(P_{A,}P_{B})=f_{1}(P_{A,}{P_{B}}^{\prime })={L_{AB}}^{2} \end{array}$$
  Because of the choice of *P*_*B*_′ = (*y*_1_′, *y*_2_′, …, *y*_*n*_′), and *r*′, it must hold that ${L_{AB}}^{2}\overset{C}{\equiv }\;L_{AB}{{}^{\prime }}^{2}$, and $R\overset{\text{C}}{\equiv }\;R^{\prime }$.
  Thus, we have
  $$\begin{array}{c} view_{1}^{\prod }(P_{A,}P_{B})\overset{C}{\equiv }\;S_{1}(P_{A},f_{1}(P_{A,}P_{B})) \end{array}$$

(2) Now, let us examine the construction of *S*_2_. Based on the inputs *P*_*B*_ = (*y*_1_, *y*_2_, …, *y*_*n*_) and *R*, *S*_2_ proceeds as follows.

1. *S*_2_ first chooses a random generator *r* and then computes $E_{P{K^{\prime }}_{A}}(2ry_{i}),i=1,...,n.$ Then, *S*_2_ chooses a point *P*_*A*_′ = (*x*_1_′, …, *x*_*n*_′) and computes $E_{PK_{A}}({x^{\prime }}_{i})$, where *i* = 1…*n*;
2. *S*_2_ performs the following calculations with the homomorphic property of the Boneh encryption algorithm:
  $$\begin{array}{c} \begin{array}{cc} {c_{0}}^{\prime } & =e(E_{P{K^{\prime }}_{A}}({x^{\prime }}_{1}),E_{P{K^{\prime }}_{A}}(2ry_{1}))...e(E_{P{K^{\prime }}_{A}}({x^{\prime }}_{n}),E_{P{K^{\prime }}_{A}}(2ry_{n}))\\ & =E{\left(-2x_{1}^{\prime }y_{1}-2x_{2}^{\prime }y_{2}-...-2x_{n}^{\prime }y_{n}\right)}^{-r} \end{array} \end{array}$$
  $$\begin{array}{c} \begin{array}{cc} {c_{1}}^{\prime } & =e(E_{P{K^{\prime }}_{A}}({x^{\prime }}_{1}),E_{P{K^{\prime }}_{A}}({x^{\prime }}_{1}))...e(E_{P{K^{\prime }}_{A}}({x^{\prime }}_{n}),E_{P{K^{\prime }}_{A}}({x^{\prime }}_{n}))\\ & =E_{P{K^{\prime }}_{A}}({x_{1}^{\prime }}^{2}+...+{x_{n}^{\prime }}^{2}) \end{array} \end{array}$$
  $$\begin{array}{c} \begin{array}{cc} c_{2} & =e(E_{PK_{A}^{\prime }}(2ry_{1}),E_{PK_{A}^{\prime }}(2ry_{1}))...e(E_{PK_{A}^{\prime }}(2ry_{n}),E_{PK_{A}^{\prime }}(2ry_{n}))\\ & =E_{PK_{A}^{\prime }}{\left({y_{1}}^{2}+...+{y_{n}}^{2}\right)}^{4r^{2}} \end{array} \end{array}$$
3. *S*_2_ computes $R^{\prime }={{c^{\prime }}_{0}}^{r}*{c^{\prime }}_{1}*c_{2}^{-4r^{2}}$.
4. *S*_2_ outputs the message list of Bob as follows.
  $$\begin{array}{c} \begin{array}{c} \left\{S_{2}(P_{B},f_{2}(P_{A,}P_{B}))\}=\{P_{B},r,E_{PK_{A}}(2rP_{B}),{c^{\prime }}_{0},{c^{\prime }}_{1},c_{2},R^{\prime }\right\} \end{array} \end{array}$$
  Note that in this protocol,
  $$\begin{array}{c} view_{2}^{\Pi }(P_{A,}P_{B})=\{P_{B},r,E_{PK_{A}}(2rP_{B}),c_{0},c_{1},c_{2},R\} \end{array}$$
  $$\begin{array}{c} f_{2}(P_{A,}P_{B})=f_{2}({P_{A}}^{\prime },P_{B})=R \end{array}$$
  Because of the choice of the point *P*_*A*_′ = (*x*_1_′, *x*_2_′, …, *x*_*n*_′), it must hold that $c_{0},c_{1}\overset{C}{\equiv }\;{c^{\prime }}_{0},{c^{\prime }}_{1},$ $R\overset{C}{\equiv }\;R^{\prime }$.
  Thus, we have
  $$\begin{array}{c} \left\{view_{2}^{\prod }(P_{A,}P_{B})\}\overset{C}{\equiv }\;\{S_{2}(P_{B},f_{2}(P_{A,}P_{B}))\right\} \end{array}$$

(3) Then, we verify the construction of *S*_3_. Based on the inputs $E_{PK_{A}}(P_{A})$, $E_{PK_{A}}(P_{B})$, *c*_0_, *c*_1_, and *c*_2_. *S*_3_ proceeds as follows.

1. *S*_3_ chooses
  $$\begin{array}{c} {P_{A}}^{\prime }=({x_{1}}^{\prime },{x_{2}}^{\prime },...,{x_{n}}^{\prime }), \end{array}$$
  $$\begin{array}{c} {P_{B}}^{\prime }=({y_{1}}^{\prime },{y_{2}}^{\prime },...,{y_{n}}^{\prime }) \end{array}$$
  and a random number *r*′. *S*_3_ computes $E_{P{K^{\prime }}_{A}}({x^{\prime }}_{i})$, $E_{P{K^{\prime }}_{A}}(2r^{\prime }{y_{i}}^{\prime })$, where *i* = 1, 2, 3…*n*.
2. *S*_3_ computes
  $$\begin{array}{c} \begin{array}{cc} {c_{0}}^{\prime } & =e(E_{P{K^{\prime }}_{A}}({x^{\prime }}_{1}),E_{P{K^{\prime }}_{A}}(2r^{\prime }y_{1}^{\prime }))...e(E_{P{K^{\prime }}_{A}}({x^{\prime }}_{n}),E_{P{K^{\prime }}_{A}}(2r^{\prime }y_{n}^{\prime }))\\ & =E{\left(-2x_{1}^{\prime }y_{1}^{\prime }-2x_{2}^{\prime }y_{2}^{\prime }-...-2x_{n}^{\prime }y_{n}^{\prime }\right)}^{-r^{\prime }} \end{array} \end{array}$$
  $$\begin{array}{c} \begin{array}{cc} {c_{1}}^{\prime } & =e(E_{P{K^{\prime }}_{A}}({x^{\prime }}_{1}),E_{P{K^{\prime }}_{A}}({x^{\prime }}_{1}))...e(E_{P{K^{\prime }}_{A}}({x^{\prime }}_{n}),E_{P{K^{\prime }}_{A}}({x^{\prime }}_{n}))\\ & =E_{P{K^{\prime }}_{A}}({x_{1}^{\prime }}^{2}+...+{x_{n}^{\prime }}^{2}) \end{array} \end{array}$$
  $$\begin{array}{c} \begin{array}{cc} {c_{2}}^{\prime } & =e(E_{PK_{A}}(2r^{\prime }y_{1}^{\prime }),E_{PK_{A}}(2r^{\prime }y_{1}^{\prime }))...e(E_{PK_{A}}(2r^{\prime }{y^{\prime }}_{n}),E_{PK_{A}}(2r^{\prime }{y^{\prime }}_{n}))\\ & =E_{PK_{A}}{\left({y_{1}^{\prime }}^{2}+...+{y_{n}^{\prime }}^{2}\right)}^{4r^{\prime 2}} \end{array} \end{array}$$
3. *S*_3_ outputs the message list of cloud
  $$\begin{array}{c} S_{3}(E(P_{A}),E(P_{B}),E(P_{A},P_{B}))=\{E_{PK_{A}}({P_{A}}^{\prime }),E_{PK_{A}}({P_{B}}^{\prime }),{c^{\prime }}_{0},{c^{\prime }}_{1},{c^{\prime }}_{2}\} \end{array}$$
  Note that in this protocol, we have
  $$view_{3}^{\Pi }(E_{PK_{A}}(P_{A}),E_{PK_{A}}(P_{B}))=\{E_{PK_{A}}(P_{A}),E_{PK_{A}}(P_{B}),c_{0},c_{1},c_{2}\}$$
  $$\begin{array}{c} f_{2}(P_{A,}P_{B})=f_{1}({P^{\prime }}_{A,}P_{B})=R \end{array}$$
  Because of the choice of the points *P*_*A*_′, *P*_*B*_′ and *r*′, it must hold that
  $$\begin{array}{c} E_{PK_{A}}(P_{A}),E_{PK_{A}}(P_{B})\overset{C}{\equiv }\;E_{P{K^{\prime }}_{A}}({P^{\prime }}_{A}),E_{P{K^{\prime }}_{A}}({P^{\prime }}_{B}), \end{array}$$
  Obviously, $c_{0},c_{1},c_{2}\overset{C}{\equiv }\;{c_{0}}^{\prime },{c_{1}}^{\prime },{c_{2}}^{\prime }$.
  Thus, we have
  $$\begin{array}{c} view_{3}^{\Pi }(P_{A},P_{B})\overset{C}{\equiv }\;S_{3}(E(P_{A}),E(P_{B}),E(P_{A},P_{B}.)) \end{array}$$
  *S*_3_ proves that the cloud server can only calculate the values from Alice and Bob using its own input and output. Because the messages that it obtains are encrypted, the cloud server cannot decrypt them. Specifically, the cloud server cannot obtain any private information from Alice or Bob.

(4) We prove the construction of *S*_4_.

When Alice conspires with the server, their inputs are

$$\begin{array}{c} P_{A},E(P_{A}),E(P_{B}),f_{1}(P_{A,}P_{B}),E(P_{A},P_{B}). \end{array}$$

Combining with the construction of *S*_1_ and *S*_3_, we simulate *S*_4_ in a similar manner. *S*_4_ obtains the message list of Alice and the cloud server.

$$\begin{array}{c} \begin{array}{c} S_{4}(P_{A},E(P_{A}),E(P_{B}),f_{1}(P_{A,}P_{B}),E(P_{A},P_{B}))\\ =\{P_{A},E_{PK_{A}}(P_{A}),E_{PK_{A}}({P^{\prime }}_{B}),{c^{\prime }}_{0},{c^{\prime }}_{1},{c^{\prime }}_{2},R^{\prime },L_{AB}{{}^{\prime }}^{2}\} \end{array} \end{array}$$

Clearly, Alice can decrypt all the ciphertexts coming from the information sequence with her own key pair. However, Alice still cannot obtain Bob’s private information through the above information. If she wants to obtain Bob’s private information, she has to solve the following problem:

$$\left\{ \begin{array}{c} D_{SK_{A}}(c_{0})={\left(\sum_{i=1}^{n}2x_{i}{y^{\prime }}_{i}\right)}^{r^{\prime }}\\ D_{SK_{A}}(c_{2})={\left({y_{1}^{\prime }}^{2}+...+{y_{n}^{\prime }}^{2}\right)}^{4{r^{\prime }}^{2}}\\ (\sum_{i=1}^{n}{x_{i}}^{2})-2\sum_{i=1}^{n}x_{i}{y^{\prime }}_{i}+(\sum_{i=i}^{n}{{y^{\prime }}_{i}}^{2})=L_{AB}{{}^{\prime }}^{2} \end{array}\right.$$

The number of equations is less than the number of unknowns when *i* ≥ 3.

The view of Alice and the server is

$$view_{4}^{\Pi }(P_{A},P_{B})=\{P_{A},E_{PK_{A}}(P_{A}),E_{PK_{A}}(P_{B}),c_{0},c_{1},c_{2},R,{L_{AB}}^{2}\}$$

Because of the choice of the point *P*_*B*_′ = (*y*_1_′, *y*_2_′, …, *y*_*n*_′), it must hold that

$$\begin{array}{c} E_{PK_{A}}(P_{B})\overset{C}{\equiv }\;E_{PK_{A}}({P_{B}}^{\prime }). \end{array}$$

Thus, we have

$$\begin{array}{c} c_{i}\overset{c}{\equiv }\;{c^{\prime }}_{i},i=0,1,2,\;\;R_{}\overset{c}{\equiv }\;{R^{\prime }}_{}. \end{array}$$

Therefore, we can obtain

$$view_{4}^{\Pi }(P_{A},P_{B})\overset{C}{\equiv }\;S_{4}(P_{A},E(P_{A})E(P_{B}),f_{1}(P_{A,}P_{B}),E(P_{A},P_{B}))$$

(5) Similarly, *S*_5_ can be constructed.

We have

$$view_{5}^{\Pi }(P_{A},P_{B})\overset{C}{\equiv }\;S_{5}(P_{B},E(P_{A})E(P_{B}),f_{2}(P_{A,}P_{B}),E(P_{A},P_{B}))$$

*S*_4_ and *S*_5_ demonstrate that even if the cloud server and the participants conspire, they cannot obtain any private information about another participant.

In conclusion, the SMC-SED protocol is privacy preserving when *n* ≥ 3.

### Performance analysis

In this section, the performance analysis of SMC-SED and other similar protocols will be discussed. For the convenience of comparison with SMC-SED, we choose protocols based on the privacy homeomorphism technique, including those documented in Refs. [28] [29] [30]. The comparison details are displayed in Table 2.

**Table 2:** Efficiency comparisons between SMC-SED with the other protocols.

Protocol	Computational Complexity	Computational Complexity for 3-dimensional vector	Communication between parties
Amirbekyan’s protocol [28]	3*nM*_*N*^2^_ + *nM*_*M*_	9*M*_*N*^2^_ + 3*M*_*M*_	2
Rane’s protocol [29]	(*n* + 1)*M*_*N*^2^_ + 2*ndM*_*E*_ + *nM*_*M*_	4*M*_*N*^2^_ + 6*dM*_*E*_ + 3*M*_*M*_	n
Huang’s protocol [30]	(*n* + 2)*M*_*N*^2^_ + 2*ndM*_*E*_ + *nM*_*M*_	5*M*_*N*^2^_ + 6*dM*_*E*_ + 3*M*_*M*_	2
SMC-SED	(2*n* + 1)*M*_*N*_ + 2*dM*_*E*_ + 2*M*_*M*_	7*M*_*N*_ + 2*dM*_*E*_ + 2*M*_*M*_	1

Communication round complexity: In SMC-SED, Alice and Bob communicate with each other one time in Step 4; thus, the communication round complexity is 1 round. Except for Rang’s protocol, whose communication cost is proportional to the original vector dimension n, all protocols have a communication efficiency of 2.

Computational complexity: We ignore the computational cost of creating random numbers and the key pair for homomorphic encryption, which can be completed in the preprocessing stage. Only the calculation phase, whose primary computational cost is a function of the dimensions and the complexity of homomorphic encryption, is considered. Let *M*_*N*_, *M*_*E*_, and *M*_*M*_ represent the homomorphic encryption, modular exponentiation and modular multiplication, respectively. Different homomorphic algorithms are adopted by different protocols. The schemes in [28], [29], [30] adopted the Paillier homomorphic algorithm, whose modular operator *p*^2^*q*^2^, while SMC-SED adopts the Boneh homomorphic algorithm, whose modular operator is *pq*. For convenience of comparison, the modular operator of SMC-SED is *M*_*N*_. The modular operator of the other schemes is *M*_*N*^2^_.

In SMC-SED, to calculate a distance with n dimensions, Alice needs to perform *n* encryptions and one decryption, i.e., $E_{PK_{A}}(x_{i})$, *i* = 1…*n*, ${L_{AB}}^{2}=D_{SK_{A}}(R)$. Bob must perform *n* encryptions, 2 modular exponentiations and 2 modular multiplications, i.e., $E_{PK_{A}}(2ry_{i})$, *i* = 1…*n*, $R={c_{0}}^{r}*c_{1}*c_{2}^{-4r^{2}}$. Therefore, the computational complexity of SMC-SED can be simplified as (2*n* + 1)*M*_*N*_ + 2*M*_*E*_ + 2*M*_*M*_ (the modular operator is N). Amirbekyan’s protocol needs 2n encryptions, n modular multiplications, and n decryptions, a total of 3*nM*_*N*^2^_ + *nM*_*M*_ (the modular operator is *N*^2^). The computational complexity of Huang’s protocol is (*n* + 2)*M*_*N*^2^_ + 2*ndM*_*E*_ + *nM*_*M*_ (modular operator is *N*^2^). Although Rane’s protocol has a similar to computational complexity to Huang’s protocol, it does not suffer an non-satisfactory communication round cost.

As observed in Table 2, SMC-SED does not possess a satisfactory computational complexity, but it achieves the lowest communication round complexity using the cloud server. However, in SMC-SED, the modular operator is *N*_1_ = *pq*, and the radix is g. In the other protocols, the modular operator is *n*_2_ = *N*_1_^2^ = *p*^2^*q*^2^, and the radix is *g*^*m*^. Because *N*_1_ ≫ *N*_2_, *g*^*m*^ > *g*, SMC-SED reduces the number of modular power operations at high orders of magnitude and reduces the users’ computing costs. Therefore, the computational complexity of SMC-SED is better than that of the other protocols.

### Experimental analysis

Here, we give a quantitative analysis for our scheme and the other protocols. The runtime environment is an Intel Core i5 @CPU at 3.2GHz with 4.00 GB of RAM. The software runtime environment is Win10 64-bit and Python 3.6. The modulus N in the homomorphic algorithms is 256, 512, 1024 and 2048 bits. The times for modular exponentiation under the different modules and different exponential sizes are listed in Table 3.

**Table 3:** Times for modular exponentiation (ms).

Module Size	256bits	512bits	1024bits	2048bits	2048bits
Exponential Size	160	160	160	160	256
Time (ms)	0.176	0.450	0.758	1.571	3.701

**Fig 2:**
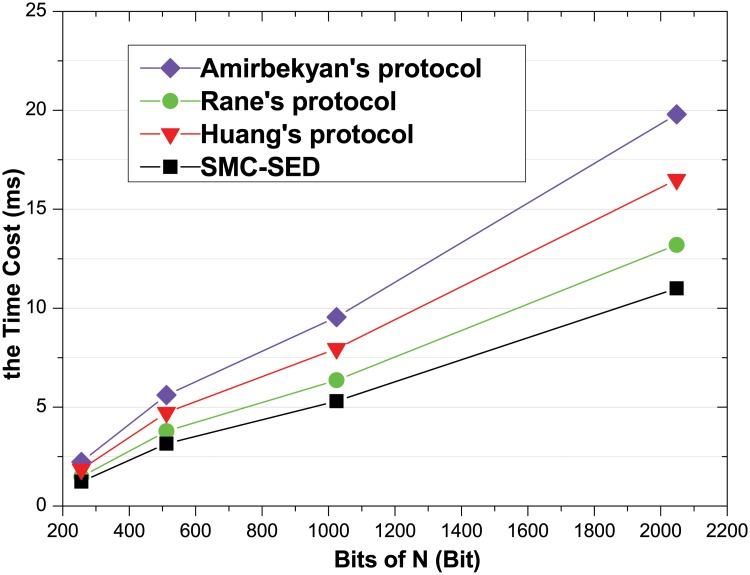
Time cost comparison.

From Table 3, we see that if the exponent size remains unchanged, the time will increase with increasing modulus, and the growth factor is approximately 2.1. According to Table 2, if we suppose that the time cost of SMC-SED is *T*_*A*_ = (2*n* + 1)*M*_*N*_, the time cost of Amirbekyan’s protocol is *T*_*A*_ ≈ 3*n* * 2.1 * *M*_*N*_, the time cost of Rane’s protocol is *T*_*R*_ ≈ (*n* + 1) * 2.1 * *M*_*N*_, and the time cost of Huang’s protocol is *T*_*A*_ ≈ (*n* + 2)*2.1*M_*N*_. Compared with *M*_*N*_, the cost of *M*_*E*_ and *M*_*M*_ is very small, and from Table 2, we can see that different protocol differ little in the cost of *M*_*E*_ and *M*_*M*_. For convenience of calculation, we ignore the time cost of the modular exponentiation and the modular multiplication. Obviously, the overall computational cost of the secure computation protocols is consistent with that of modular exponentiation. Assume that the exponent size is 160, N is 256, 512, 1024 or 2048 and *n* = 3. We compare the trend of the time cost of the different protocol (see Fig 2). The horizontal axis is the bits of N, and the vertical axis is the time cost (ms).

**Fig 3:**
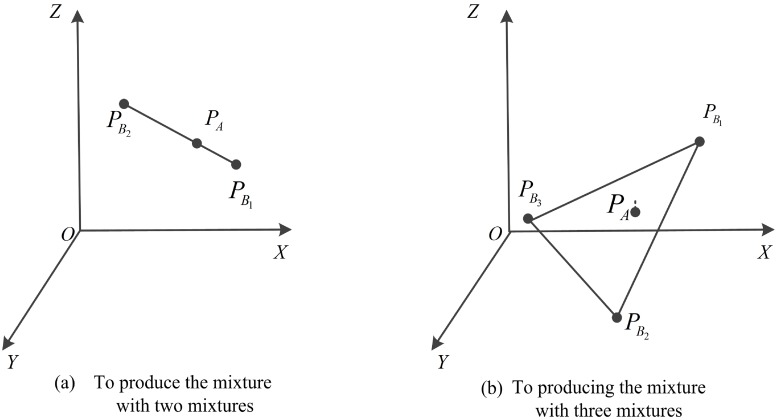
The output of the mixtures.

It can be observed from Fig 3 that the actual experimental simulation is completely consistent with the theoretical analysis. SMC-SED indeed reduces the computing time for the users compared with the protocol in [28–30]. Therefore, SMC-SED is effective and efficient in a practical environment.

## Solutions

In this section, we use SMC-SED as the basic subprotocol, and we solve the problems of the triangle area and volume calculations and give an extend protocol about the point-line distance.

### SMC-TA

The problem of SMC-TA is as follows: Alice has a point *P*_*A*_ = (*x*_*A*_, *y*_*A*_, *z*_*A*_), and Bob has two points $P_{B_{1}}=(x_{B_{1}},y_{B_{1}},z_{B_{1}}),P_{B_{2}}=(x_{B_{2}},y_{B_{2}},z_{B_{2}})$. These three points can form a triangle in space. Alice and Bob want to know the area of the triangle without disclosing their private messages. To solve this problem, we first calculate the square of the Euclidean distances ${L_{AB_{1}}}^{2}$ and ${L_{AB_{2}}}^{2}$, and then, we convert the problem of a triangle area into a determinant calculation about distances. The details of the protocol are as follows.

**Protocol 2**: Secure multiparty computation of the triangle area

**Input**: Alice’s input is *P*_*A*_ = (*x*_*A*_, *y*_*A*_, *z*_*A*_), and Bob’s inputs are $P_{B_{1}}=(x_{B_{1}},y_{B_{1}},z_{B_{1}}),$ $P_{B_{2}}=(x_{B_{2}},y_{B_{2}},z_{B_{2}}).$

**Output**: The area $S_{\Delta AB_{1}B_{2}}$ of the triangle is formed by *P*_*A*_, $P_{B_{1}},P_{B_{2}}.$

Step 1 Alice and Bob compute ${L_{AB_{1}}}^{2}$, ${L_{AB_{2}}}^{2}$ using SMC-SED. In addition, Alice obtain ${L_{AB_{1}}}^{2}$, ${L_{AB_{2}}}^{2}$.
Step 2 Bob computes ${L_{B_{1}B_{2}}}^{2}$ and sends it to Alice.
Step 3 Alice computes
  $$\begin{array}{c} S_{\Delta AB_{1}B_{2}}=\sqrt{\;\;\;\;(\;(\frac{1}{4})\cdot 2\cdot {\left(\frac{-1}{2}\right)}^{3}\cdot A)} \end{array}$$
  where
  $$\begin{array}{c} A=|\begin{array}{cccc} 0 & 1 & 1 & 1\\ 1 & 0 & {L^{2}}_{AB_{1}} & {L^{2}}_{AB_{2}}\\ 1 & {L^{2}}_{AB_{1}} & 0 & {L^{2}}_{B_{1}B_{2}}\\ 1 & {L^{2}}_{AB_{2}} & {L^{2}}_{B_{1}B_{2}} & 0 \end{array}| \end{array}$$
  Then, Alice sends the result to Bob.

### SMC-TV

Similar to protocol 2, if Alice has a point *P*_*A*_ = (*x*_*A*_, *y*_*A*_, *z*_*A*_) and Bob has three points $P_{B_{1}}=(x_{B_{1}},y_{B_{1}},z_{B_{1}})$, $P_{B_{2}}=(x_{B_{2}},y_{B_{2}},z_{B_{2}})$, $P_{B_{3}}=(x_{B_{3}},y_{B_{3}},z_{B_{3}})$. These four points form a tetrahedron in space. Alice and Bob want to know the volume of a tetrahedron without disclosing their private information. We call this problem the secure multiparty computation of the volume of a tetrahedron.

**Protocol 3**: Secure multiparty computation of the tetrahedron volume

**Input**: Alice’s input is *P*_*A*_, and Bob’s inputs are $P_{B_{1}}$, $P_{B_{2}}$, $P_{B_{3}}$;

**Output**: The volume $V_{AB_{1}B_{2}B_{3}}$ of the tetrahedron is formed by *P*_*A*_, $P_{B_{1}}$, $P_{B_{2}}$, $P_{B_{3}}$

Step 1 Using SMC-ED, Alice and Bob compute
  $$\begin{array}{c} {L_{AB_{1}}}^{2}=D(P_{A},P_{B_{1}}), \end{array}$$
  $$\begin{array}{c} {L_{AB_{2}}}^{2}=D(P_{A},P_{B_{2}}), \end{array}$$
  $$\begin{array}{c} {L_{AB_{3}}}^{2}=D(P_{A},P_{B_{3}}). \end{array}$$
  In addition, Alice gets the results.
Step 2 Bob computes ${L_{B_{1}B_{2}}}^{2}$, ${L_{B_{1}B_{3}}}^{2}$, ${L_{B_{2}B_{3}}}^{2}$ and sends them to Alice;
Step 3 Alice computes
  $$\begin{array}{c} \begin{array}{ccc} & V_{AB_{1}B_{2}B_{3}}= & \sqrt{\frac{1}{36}\cdot 2\cdot {\left(\frac{-1}{2}\right)}^{4}\cdot |\begin{array}{ccccc} 0 & 1 & 1 & 1 & 1\\ 1 & 0 & L^{2}AB_{1} & L^{2}AB_{2} & L^{2}AB_{3}\\ 1 & L^{2}AB_{1} & 0 & L^{2}B_{1}B_{2} & L^{2}B_{1}B_{3}\\ 1 & L^{2}AB_{2} & L^{2}B_{1}B_{2} & 0 & L^{2}B_{2}B_{3}\\ 1 & L^{2}AB_{3} & L^{2}B_{1}B_{3} & L^{2}B_{2}B_{3} & 0 \end{array}|} \end{array} \end{array}$$
  and sends the result to Bob.

### SMC-DPL

Using the SMC-TA as a building block, we further solve the problem of the secure multiparty computation of a point and a line. Specifically, Alice has a private point *P*_*A*_ = (*x*_*A*_, *y*_*A*_, *z*_*A*_), and Bob has a private line

$$\begin{array}{c} L_{}:\{\begin{array}{c} A_{1}x+B_{1}y+C_{1}z+D_{1}=0\\ A_{2}x+B_{2}y+C_{2}z+D_{2}=0 \end{array} \end{array}$$

Alice and Bob want to know the distance between the point and the line without disclosing their private information.

We know that the area of the triangle equals half the base of the triangle times the height. If we can determine the area of the triangle using SMC-TA and the base of the triangle, the height of the triangle is exactly the distance between the point and the line. It is easy for us to obtain the distance as follows.

**Protocol 4**: Secure multiparty computation of the point-line distance

**Input**: Alice’s input is *P*_*A*_ = (*x*_*A*_, *y*_*A*_, *z*_*A*_). Bob’s input is

$$\begin{array}{c} L_{}:\{\begin{array}{c} A_{1}x+B_{1}y+C_{1}z+D_{1}=0\\ A_{2}x+B_{2}y+C_{2}z+D_{2}=0 \end{array} \end{array}$$

**Output**: The distance between *P*_*A*_ and *L*.

Step 1 Bob randomly chooses $P_{B_{1}}=(x_{B_{1}},y_{B_{1}},z_{B_{1}}),$ $P_{B_{2}}=(x_{B_{2}},y_{B_{2}},z_{B_{2}})$ on line L. Bob computes the distance between these two points, defined as ${L^{}}_{B_{1}B_{2}}$.
Step 2 Alice and Bob use SMC-TA to compute the area $S_{\Delta AB_{1}B_{2}}$ privately.
Step 3 Bob computes $h=\frac{2S_{\Delta AB_{1}B_{2}}}{{L^{}}_{B_{1}B_{2}}}$ with $S_{\Delta AB_{1}B_{2}}$ and ${L^{}}_{B_{1}B_{2}}$. Then, Bob sends the result to Alice.

The security of the protocol follows from Theorem 1.

In the same way as [6], if we use the solution of SMC-TV as a building block, we can also solve other geometric security problems such as point-line, point-surface, and line-line problems.

### Security

Theorem 2. In the semi-honest model, SMC-TA is private.

Proof: The protocol security is that the parties cannot use the intermediate results to obtain private information about each other. In our protocol, Alice obtains ${L_{AB_{1}}}^{2}$, ${L_{AB_{2}}}^{2}$, and ${L_{B_{1}B_{2}}}^{2}$. However, if Alice wants to obtain Bob’s private information, she has to find two points on the concentric circle with radii ${L^{}}_{AB_{1}}$ and ${L^{}}_{AB_{2}}$, where the distance between these two points is ${L^{}}_{B_{1}B_{2}}$. Specifically, Alice must solve the following problem:

$$\left\{ \begin{array}{c} {\left(x_{A}-x{}_{B_{1}}\right)}^{2}+{\left(y_{A}-y{}_{B_{1}}\right)}^{2}+{\left(z_{A}-z{}_{B_{1}}\right)}^{2}={L_{AB_{1}}}^{2}\\ {\left(x_{A}-x{}_{B_{2}}\right)}^{2}+{\left(y_{A}-y{}_{B_{2}}\right)}^{2}+{\left(z_{A}-z{}_{B_{2}}\right)}^{2}={L_{AB_{2}}}^{2}\\ {\left(x_{B_{1}}-x{}_{B_{2}}\right)}^{2}+{\left(y_{B_{1}}-y{}_{B_{2}}\right)}^{2}+{\left(z_{B_{1}}-z{}_{B_{2}}\right)}^{2}={L_{B_{1}B_{2}}}^{2} \end{array}\right.$$

Clearly, there are three equations. The number of equations is less than the number of unknowns. Therefore, it is difficult for Alice to obtain Bob’s private information. On the other side, Bob has ${L_{B_{1}B_{2}}}^{2},S_{\Delta AB_{1}B_{2}},$ and he can calculate the height of the triangle according to the formula for the area of a triangle $S_{\Delta AB_{1}B_{2}}=\frac{1}{2}{L^{}}_{B_{1}B_{2}}\cdot h.$ However, Bob can only extrapolate the potential location of *P*_*A*_ and not obtain the exact location.

The construction of simulators of the proof is similar to that of Theorem 1. Therefore, we construct the simulators *S*_1_, *S*_2_, *S*_3_, *S*_4_, and *S*_5_ as follows.

1. The construction of the simulator *S*_1_
  Based on *P*_*A*_ and $S_{\Delta AB_{1}B_{2}}$, *S*_1_ chooses two points ${P_{B_{1}}}^{\prime }=({x_{B_{1}}}^{\prime },{y_{B_{1}}}^{\prime },{z_{B_{1}}}^{\prime })$, ${P_{B_{2}}}^{\prime }=({x_{B_{2}}}^{\prime },{y_{B_{2}}}^{\prime },{z_{B_{2}}}^{\prime })$ and computes ${L_{AB_{1}^{\prime }}}^{2}$,${L_{AB_{2}^{\prime }}}^{2}$,${L_{B_{1}^{\prime }B_{2}^{\prime }}}^{2}$. Then, *S*_1_ computes $S_{\Delta A{B^{\prime }}_{1}{B^{\prime }}_{2}}$ by the Cayley-Menger determinant. *S*_1_ obtains the following list:
  $$\begin{array}{c} \begin{array}{ccc} & S_{1}(P_{A},f_{A}(P_{A,}P_{B}))= & \left\{P_{A},{L_{AB_{1}^{\prime }}}^{2},{L_{AB_{2}^{\prime }}}^{2},{L_{B_{1}^{\prime }B_{2}^{\prime }}}^{2},S_{\Delta A{B^{\prime }}_{1}{B^{\prime }}_{2}}\right\} \end{array} \end{array}$$
  Note that in this protocol, we have
  $$\begin{array}{c} \begin{array}{ccc} & view_{1}^{\Pi }(P_{A},P_{B})= & \left\{P_{A},{L_{AB_{1}}}^{2},{L_{AB_{2}}}^{2},{L_{B_{1}B_{2}}}^{2},S_{\Delta AB_{1}B_{2}}\right\} \end{array} \end{array}$$
  Because of the choice of the points ${P_{B_{1}}}^{\prime }$, ${P_{B_{2}}}^{\prime },$ it must hold that
  $$\begin{array}{c} \begin{array}{cccc} & {L_{AB_{1}^{\prime }}}^{2}\overset{C}{\equiv }\;{L_{AB_{1}}}^{2} & {L_{AB_{2}^{\prime }}}^{2}\overset{C}{\equiv }\;{L_{AB_{2}}}^{2}\\ & {L_{B_{1}^{\prime }B_{2}^{\prime }}}^{2}\overset{C}{\equiv }{L_{B_{1}B_{2}}}^{2} & S_{\Delta A{B^{\prime }}_{1}{B^{\prime }}_{2}}\overset{C}{\equiv }\; & S_{\Delta AB_{1}B_{2}} \end{array} \end{array}$$
  Thus, we have
  $$\begin{array}{c} \left\{view_{1}^{\Pi }(P_{A,}P_{B})\}\overset{C}{\equiv }\{S_{1}(P_{A},f_{A}(P_{A,}P_{B}))\right\} \end{array}$$
2. Similarly, we can construct the other four simulators *S*_2_, *S*_3_, *S*_4_, and *S*_5_ and obtain the following formula.
  $$\begin{array}{c} \begin{array}{c} \left\{view_{2}^{\Pi }(P_{A,}P_{B})\}\overset{C}{\equiv }\{S_{2}(P_{B},f_{B}(P_{A,}P_{B}))\right\} \end{array} \end{array}$$
  $$\begin{array}{c} \begin{array}{cc} & \;view_{3}^{\Pi }(P_{A},P_{B})\;\;\overset{C}{\equiv }S_{3}(E(P_{A}),E(P_{B}),E(P_{A},P_{B}))\;\;\;\; \end{array} \end{array}$$
  $$\begin{array}{c} \begin{array}{c} view_{4}^{\Pi }(P_{A},P_{B})\overset{C}{\equiv }\;S_{4}(P_{A},E(P_{A})E(P_{B}),f_{1}(P_{A,}P_{B}),E(P_{A},P_{B})) \end{array} \end{array}$$
  $$\begin{array}{c} \begin{array}{c} view_{5}^{\Pi }(P_{A},P_{B})\overset{C}{\equiv }\;S_{5}(P_{B},E(P_{A})E(P_{B}),f_{2}(P_{A,}P_{B}),E(P_{A},P_{B})) \end{array} \end{array}$$

Theorem 3. In the semi-honest model, SMC-TV, denoted by Π, is private.

Proof: In our protocol, Alice knows ${L_{AB_{1}}}^{2}$, ${L_{AB_{2}}}^{2}$, ${L_{AB_{3}}}^{2}$, ${L_{B_{1}B_{2}}}^{2}$, ${L_{B_{1}B_{3}}}^{2}$, and ${L_{B_{2}B_{3}}}^{2}$, which are the squares of the six sides of the tetrahedron. However, if Alice wants to obtain Bob’s private information, she has to find a triangle on the concentric sphere with radii ${L_{AB_{1}}}^{2}$, ${L_{AB_{2}}}^{2}$, ${L_{AB_{3}}}^{2}$, where the lengths of the three sides of this triangle are $L_{B_{1}B_{2}}$, $L_{B_{1}B_{3}}$, and $L_{B_{2}B_{3}}$. Thus, Alice should solve the following problem:

$$\left\{ \begin{array}{c} {\left(x_{A}-x{}_{B_{1}}\right)}^{2}+{\left(y_{A}-y{}_{B_{1}}\right)}^{2}+{\left(z_{A}-z{}_{B_{1}}\right)}^{2}={L_{AB_{1}}}^{2}\\ {\left(x_{A}-x{}_{B_{2}}\right)}^{2}+{\left(y_{A}-y{}_{B_{2}}\right)}^{2}+{\left(z_{A}-z{}_{B_{2}}\right)}^{2}={L_{AB_{2}}}^{2}\\ {\left(x_{A}-x{}_{B_{3}}\right)}^{2}+{\left(y_{A}-y{}_{B_{3}}\right)}^{2}+{\left(z_{A}-z{}_{B_{3}}\right)}^{2}={L_{AB_{3}}}^{2}\\ {\left(x_{B_{1}}-x{}_{B_{2}}\right)}^{2}+{\left(y_{B_{1}}-y{}_{B_{2}}\right)}^{2}+{\left(z_{B_{1}}-z{}_{B_{2}}\right)}^{2}={L_{B_{1}B_{2}}}^{2}\\ {\left(x_{B_{2}}-x{}_{B_{3}}\right)}^{2}+{\left(y_{B_{2}}-y{}_{B_{3}}\right)}^{2}+{\left(z_{B_{2}}-z{}_{B_{3}}\right)}^{2}={L_{B_{2}B_{3}}}^{2}\\ {\left(x_{B_{1}}-x{}_{B_{3}}\right)}^{2}+{\left(y_{B_{1}}-y{}_{B_{3}}\right)}^{2}+{\left(z_{B_{1}}-z{}_{B_{3}}\right)}^{2}={L_{B_{1}B_{3}}}^{2} \end{array}\right.$$

Clearly, the number of equations is less than the number of unknowns. Alice cannot determine Bob’s private information. On the other hand, Bob knows ${L_{B_{1}B_{2}}}^{2}$, ${L_{B_{1}B_{3}}}^{2}$, ${L_{B_{2}B_{3}}}^{2}$ and $V_{AB_{1}B_{2}B_{3}}$. According to the relation between the area of a triangle and the volume of a tetrahedron, Bob can calculate the height of the tetrahedron. However, he cannot determine the exact location of *P*_*A*_. The construction of the simulators in theorem 3 is similar to that of theorem 2.

Theorem 4: The protocol for computing the point-line distance is private.

Proof:Protocol 4 is an extension of protocol 2; thus, its privacy is determined by Theorem 2.

## Complexity and comparison

### Complexity

Computational complexity: In SMC-TA, two parties utilize cooperation twice using SMC-SED. Alice and Bob can send the initial data to the cloud server once. In addition, the cloud server delivers the results to Bob when finishing its computation. In Step 2, Bob performs the normal addition operation 3 times and the normal multiplication operation 2 times. In Step 3, Alice performs a fourth-order matrix operation one time, the normal multiplication operation 3 times and the exponentiation operation one time. If we ignore the ordinary operations, the computational complexity of our protocol is 11*M*_*N*_ + 3*M*_*E*_ + 2*M*_*M*_. In SMC-TV, Bob computes the square of the Euclidean distance 3 times. Alice performs a fifth-order matrix operation one time, the common multiplication operation 3 times and the common exponentiation operation one time. Thus, the computational complexity of SMC-TV is 15*M*_*N*_ + 6*M*_*E*_ + 4*M*_*M*_. The computational complexity of SMC-DPL is the same as that of SMC-TA.

Communication complexity: In SMC-TA, Bob can keep the results of the distance calculation and send them ${L^{2}}_{B_{1}B_{2}}$ to Alice in Step 2. Alice sends $S_{\Delta AB_{1}B_{2}}$ to Bob in Step 3. Thus, there are 2 rounds between Alice and Bob in our protocol. In the same way, in SMC-TV, the communication between two participants consists of 2 rounds. The communication complexity of SMC-DPL is the same as that of SMC-TA.

### Comparison

This section provides comparisons of the complexity and performance of this protocol with the schemes in references [6]and [25]. We show the comparison of the complexity of our protocol with that of [6] in Table 4. In Table 5, we compare the performance of our protocol with the protocols in [6] and [25].

**Table 4:** Complexity comparison between our scheme with the scheme in [6].

Problem	Protocol	Computational Complexity	Communication between parties
Triangular Area	Protocol 2	11*M*_*N*_ + 3*M*_*E*_ + 2*M*_*M*_	2
Tetrahedral Volume	Protocol 3	15*M*_*N*_ + 6*M*_*E*_ + 3*M*_*M*_	2
	*Ref*. [6]	9*M*	2

**Table 5:** Performance comparison of our scheme and schemes in [6, 25].

Protocol	Triangular Area	Tetrahedral Volume	Point-Line Distance	Point-Plane Distance	Line-Plane Relationship	Plane-Plane Relationship	Cloud Platform
*Ref*. [6]	N	Y	N	Y	Y	Y	N
*Ref*. [25]	N	N	Y	Y	N	N	Y
*Ours*	Y	Y	Y	Y	Y	Y	Y

Complexity: Because the spatial location problems involved in the literature are not identical, to perform the comparison, we chose the volume protocol in [6] to make a comparison. In [6], Bob performs third-order matrix operations 4 times, and Alice performs common multiplication calculations 5 times. Thus, the computational complexity is 9*M*, where *M* represents the number of common multiplications. The computational complexity can be neglected. Our schemes use a homomorphic cipher algorithm to protect the user’s private information, and the encryption and decryption calculations need more time. From Table 4, we can see that our schemes have the same communication complexity as the protocol in [6]; the computational complexity of our schemes is not optimal.

Performance: In [6], Li et al. studied security geometry problems such as tetrahedral volumes, point-line distances, line-plane relationships and plane-plane relationships. However, the schemes in [6] did not concern problems about triangular areas and point-line distances. In [25], the protocol was extended with the help of a cloud platform. However, the protocol can only solve the point-line distance problem and the point-plane distance problem. In contrast, this paper solves the above six problems, i.e. the triangular area, tetrahedral volume, point-line distance, point-plane distance, line-plane relationship and plane-plane relationship problems, in the same way with the cloud platform. From Table 5, we can see that the solutions in [25] have the worst performance, and their application is limited. The method in [6] is not suitable for cloud platforms. Our schemes achieve the best performance.

From the above, the complexity of our solutions is not the most satisfactory, but our schemes represent a new technique for solving secure multi-party solid geometry computation problems and can be used to solve a wider range of problems while maintaining the same level of security and being more universal.

## Application

To illustrate our motivation in developing these solutions, we present the following interesting scenario inspired by [6]: Bob has a mixture *ξ*_1_ that contains 10% of component *M*_1_, 25% of component *M*_2_ and 10% of component *M*_3_ and another mixture *ξ*_2_ that contains 2% of component *M*_1_, 11% of component *M*_2_ and 4% of component *M*_3_. Additionally, assume that Alice needs a mixture that contains 6% of component *M*_1_, 20% of component *M*_2_ and 3% of component *M*_3_. Alice wants to know if she can produce this mixture from the two mixtures that Bob has, but she does not want to disclose her needs. Similarly, Bob does not want to disclose the contents of his mixtures. If we represent the mixtures *ξ*_1_ and *ξ*_2_ by points in three-dimensional space, namely, by $P_{B_{1}}$, $P_{B_{2}}$ (see Fig 3), we can produce the mixtures represented by any point on the line segment $P_{B_{1}}$, $P_{B_{2}}$ by mixing *ξ*_1_ and *ξ*_2_ at various ratios. Thus, privately determining whether Alice can produce her mixture from Bob’s two mixtures can be reduced to privately determining whether the point that represents Alice’s mixture is on the line segment $P_{B_{1}}$, $P_{B_{2}}$. This computational geometry problem is called the point-inclusion problem.

How does one solve this problem? First, Alice and Bob use Protocol 1 to privately compute ${L^{2}}_{AB_{1}}$ and ${L^{2}}_{AB_{2}}$, and they use Protocol 2 to privately compute $S_{\Delta AB_{1}B_{2}}$. Then, Alice and Bob use Protocol 4 to privately determine *d*, the distance between point *P*_*A*_, which represents Alice’s mixture in three-dimensional space, and the line $P_{B_{1}}P_{B_{2}}$, determined by the two points that represent Bob’s two mixtures. If *d* ≠ 0, Alice cannot produce her mixture from the two mixtures that Bob has; otherwise, if *d* = 0, which implies that point *P*_*A*_ is on the line determined by the two points that represent Alice’s two mixtures, then Alice and Bob can negotiate to project these points onto a line and to privately determine whether the projection of *P*_*A*_ is inside the projection of the line segment $P_{B_{1}}P_{B_{2}}$. If the projection of *P*_*A*_ is inside that of line segment $P_{B_{1}}P_{B_{2}}$, Alice can produce her mixture from Bob’s two mixtures; otherwise, Alice cannot produce her mixture from his mixtures. In this way, Alice and Bob can solve this problem while keeping their mixtures private.

If Alice has three mixtures, Alice and Bob first use Protocol 1 to privately compute ${L^{2}}_{AB_{1}}$, ${L^{2}}_{AB_{2}}$ and ${L^{2}}_{AB_{3}}$, and they use Protocol 3 to privately compute $V_{AB_{1}B_{2}B_{3}}$. Then, Alice and Bob use Protocol 2 in [6] to determine the distance between point *P*_*A*_, which represents Alice’s mixture in three-dimensional space, and the line $P_{B_{1}}$, $P_{B_{2}}$, and $P_{B_{3}}$ is determined by the three points that represent Bob’s three mixtures (see Fig 3). If there are more than three components of interest in the mixtures, a similar analysis can be performed using a higher dimensional space.

## Conclusion

With the emergence and widespread use of cloud computing, solving the problem of multi-party computing with cloud platforms has become a new research direction. The introduction of cloud computing resources has lead to changes in the secure multi-party computing model and solutions. However, the computational task of spatial geometry calculations in traditional problems is completed via mutual interactions between two parties. Therefore, it is difficult for these protocols to used in untrusted cloud computing environments. In this paper, we propose a more general solution to solve spatial geometry security problems using a cloud platform. First, we transform the problems into the calculation of a distance. Then, we design a security protocol to solve for the Euclidean distance. Based on the above protocol, we solve two problems concerning the calculations of triangular areas and tetrahedron volumes. We prove that our protocols can resist collusion between the parties and the untrusted cloud platform, and it effectively protects the users’ privacy. In addition, we note that the proposed protocol can be used for the calculation of spatial distances such as point-line, point-surface, and line-line distance. Because of the transformation of the problems, we can solve more spatial geometry security problems with a cloud platform. However, the complexity of the protocol should be improved. Therefore, as future work, we will attempt to reduce the computational complexity of the protocol.
